# Early Detection of Porcine Reproductive and Respiratory Syndrome Virus Outbreak: Combination of Methods

**DOI:** 10.3390/vetsci12121198

**Published:** 2025-12-15

**Authors:** Cunshuai Gao, Yunzhou Wang, Mengmeng Liu, Haotian Yang, Wenjing Jiao, Xuanpan Ding, Yuan Zhao, Honggang Fan

**Affiliations:** 1 Heilongjiang Key Laboratory for Laboratory Animals and Comparative Medicine, College of Veterinary Medicine, Northeast Agricultural University, Harbin 150030, China; 2 Department of Veterinary Medicine, Shandong Vocational Animal Science and Veterinary College, Weifang 261061, China; 3 Qingdao Smart Village Development Service Center, Qingdao 266000, China; 4 Branch of Animal Husbandry and Veterinary of Heilongjiang, Academy of Agricultural Sciences, Qiqihar 161005, China

**Keywords:** PRRSV, early detection, EWMA, sow, daily indication

## Abstract

The current application of the production data exponentially weighted moving average (EWMA) model can detect PRRSV outbreaks earlier than that of processing fluid (PF) testing; however, its advantages have not been fully reported. This study aimed to analyze various production parameters, including abortion, off-feed, low appetite, and dead sows, on a daily basis following a PRRSV outbreak in an II-vx sow farm. The EWMA method was employed and the results were compared with the early detection of positive PF results. Differences in daily abnormal indicators across the three PRRSV status periods were analyzed. Additionally, this study evaluated the PRRSV detection rates in different sample types (AF, OS, and TBS) from aborted sows and compared the detection rates of different sample combinations using statistical tests. The 187-day study revealed that the first true positive (TP) alarm point for daily abortion sows occurred on day 107 and for off-feed sows on day 110. In contrast, the first RT-qPCR-positive result for PF was obtained on Day 122. The average values of daily abortions and off-feed sows in status I-A were significantly higher than those in status II-vx and I-B. Conversely, the average value of low appetite in status I-A was significantly lower than that in statuses II-vx and I-B. No significant differences were observed in the daily number of dead sows among the three groups. The RT-PCR detection rates varied significantly (*p* < 0.01) among the different sample types (AF, 43.04%; TBS, 65.82%; and OS, 74.68%), with amniotic fluid (AF) showing the lowest detection rate. Combining AF and oropharyngeal swabs (OS) samples yielded a higher detection rate than combining AF and TBS samples. Using the EWMA to monitor the daily number of aborted sows was effective for the early detection of PRRSV outbreaks.

## Simple Summary

Porcine reproductive and respiratory syndrome virus (PRRSV) has spread globally and has caused substantial economic losses in the swine industry. The use of the exponentially weighted moving average (EWMA) analysis of production parameters for the early detection of PRRSV outbreaks is a practical approach. However, there are few studies on the combination of monitoring breeding herds daily using EWMA with molecular diagnosis for early detection of PRRSV outbreaks. This study evaluated the true-positive (TP) alarm time points of different daily production indicators according to the data before and after the outbreak by utilizing EWMA in a PRRSV status II-vx sow farm and compared it with that of a processing fluid (PF) turning positive. We also compared the changes in EWMA values at different PRRSV statuses, as well as the nucleic acid detection rates of different samples from aborting sows, including amniotic fluid (AF), oropharyngeal swabs (OS), and tail blood swabs (TBS). The combination of monitoring breeding herd daily abortion using EWMA with RT-qPCR testing using AF and OS samples could be an eligible combination for early detection of PRRSV outbreaks. This will provide a more advanced method for predicting PRRSV outbreaks, which will help reduce the substantial economic losses in PRRSV management.

## 1. Introduction

Porcine reproductive and respiratory syndrome (PRRS) is induced by the PRRS virus (PRRSV), which was initially reported by Keffaber in 1989 [1]. Over the past three decades, PRRSV has spread globally, exerting a substantial economic impact on the swine industry [2]. Numerous sow farms worldwide have been infected with PRRSV in breeding herds, incurring substantial economic losses owing to its significant influence on productivity [3]. This includes an increase in abortions, pre-weaning mortality, and neonatal losses [4]. A PRRSV monitoring system was established to monitor the transmission frequency of PRRSV across different phases. Conventional PRRSV monitoring systems typically collect serum samples to detect nucleic acids via the Polymerase Chain Reaction (PCR) or antibodies using an Enzyme-Linked Immunosorbent Assay (ELISA) [5]. Recently, several studies have reported the use of new sample types for PRRSV monitoring in sow farms, such as processing fluid (PF) [6], tongue tip fluid (TTF) [7], oral fluid (OF) [8], and family oral fluid (FOF) [9]. In addition to diagnosing and surveying PRRSV outbreaks, the applicability of monitoring production indicators was also explored [10]. Weekly tracking and analysis of production data have been reported as crucial approaches for systematically monitoring the health status and productivity of breeding herds [11]. The number of sows with anorexia and abortions in the breeding herd was affected by PRRSV infection. In cases where PRRSV exhibits high pathogenicity, it can lead to the death of the sows.

Statistical process control (SPC) charts are widely employed in industrial processes and for monitoring deviations in daily production data, such as feed consumption and water intake [12]. The exponentially weighted moving average (EWMA) control method, which is part of SPC, is utilized to monitor minor fluctuations in the production process. A study indicated that using EWMA to monitor the number of weekly aborted sows associated with a PRRSV outbreak could detect it up to 4 weeks earlier than when it was recorded in the Morrison’s Swine Health Monitoring Project database [5]. However, there have been few reports on the application of EWMA to monitor PRRSV outbreaks on a daily basis using reproductive parameters.

A comparative statistical analysis study demonstrated that geometric means of weekly abortion per 1000 sows (ABTHS) in PRRSV positive stable status was 0.78 (95% confidence interval (CI) 0.59–1.02) and that in positive unstable status was 0.84 (95% CI 0.65–1.09) by using a total of 1997 weekly data including 741 unstable weeks and 1256 stable weeks from 35 monitored farms in Spain [13]. In a PRRSV-stable sow farm, the number of weekly abortions has proven to be one of the most efficient parameters for the early detection of PRRSV outbreaks [5]. A published study analyzing 23 sow farms located in the Hubei Province of China showed that the monthly sow death rate was significantly different between PRRSV-positive stable and unstable farms, which were 7.06% and 7.94%, respectively [14].

In sows experiencing reproductive failure, it is recommended to collect samples of fetal thymus and lung tissue, as well as the serum of the aborting sow, for the detection of PRRSV RNA [2]. Pooled organs, along with umbilical cords from aborted fetuses, mummified fetuses [15], and stillborn piglets from 10 sow herds were used to detect PRRSV RNA using RT-qPCR to explore the prevalence of PRRSV in Thailand [16]. The objective of this study was to assess the feasibility of the early detection of PRRSV outbreaks using EWMA for daily monitoring of production data, including the number of sows with abortion, off-feed, low appetite, and death in a sow farm. Three types of samples were collected separately from each aborted sow to optimize a combination of sample types that maximized the detection rate of PRRSV and improved the success rate of early monitoring. The key novelty of this study was the use of daily abnormal production data by EWMA combined with molecular diagnosis of abnormal sows for the early detection of PRRSV outbreaks, instead of weekly production data.

## 2. Materials and Methods

### 2.1. Basic Information and PRRSV Status of the Farm

The study was conducted on a sow farm with 2400 sow inventory located in Hunan Province, China, which is a breed-to-wean operating system with weekly batch production. The PRRS vaccination scheme in the sow herd was 4 times per year with blanket vaccination using the PRRS-modified live virus (MLV) vaccine (R98 strain). The processing fluid was routinely collected and presented as consecutive PRRSV-negative samples by RT-qPCR for at least six months. In addition, amniotic fluid (AF) samples from aborted sows were collected to test for PRRSV by RT-qPCR, which were also continuously negative during that period. Therefore, the farm was confirmed as a PRRSV positive stable category (II-vx) according to American Association of Swine Veterinarians (AASV) classification [17] from 23 October 2023, to 5 February 2024. Serum samples were collected from 78 sows for PRRSV antibody testing using the IDEXX PRRS 2XR Ab test kit on 14 November 2023, with an antibody positivity rate of 71.79% and average S/*p* value of 0.77. In breeding herds, abortions were recorded daily using Excel. The NACD30-like strain of PRRSV was first detected by AF in an aborted sow on 6 February 2024, when the PRRSV I-A status of this farm was defined. Combined with the positive results and increased number of abortions, the sow farm was identified as a PRRSV outbreak. The farm was returned to status I-B from 2 March 2024, to 26 April 2024, and then transferred to status II-vx according to the AASV classification [3]. All animal experimental procedures were approved by the Laboratory Animal Research Ethics Committee of Northeast Agricultural University (SRM-11).

### 2.2. Daily Production Data and Samples Collection

Daily production data of the breeding herd were recorded from 23 October 2023, to 26 April 2024. The study lasted 187 days. Daily production parameters, including abortion, off-feed, low appetite, and dead sows, were recorded in an Excel chart.

PF samples were collected weekly for PRRSV monitoring, and AF samples were collected from abortion sows. After the NADC30-like strain was introduced into the breeding herd, three types of samples were simultaneously collected from all aborted sows: AF, oropharyngeal swab (OS), and tail blood swab (TBS). PF samples were collected from 3-day-old piglets as previously described [18].

AF samples were collected once an abortion occurred using two short cotton swabs (Xindi Co., Wuhan, China), one to wipe the surface of fetal membranes, especially in leisure areas, and the other to wipe the skin of no less than half of the stillborn fetuses. The two cotton swabs were eluted with 1 mL saline solution.

The OS sample was collected using a 55 cm plastic shaft with a flocked polyester fiber head. It was gnawed by the sow without restraint at the beginning and then moved back and forth to the tonsil at an upward angle. The head was eluted with 1 mL saline solution.

The TBS was sampled using a sterile needle and cotton swab. The tail skin of each sow was punctured using a needle until bleeding occurred. Blood was absorbed as much as possible using a cotton swab. The cotton swab was eluted with 1 mL of saline solution. Samples, stored in a box with ice packs after collection, were delivered to the local lab within six hours.

### 2.3. RNA Extraction and RT-qPCR Test

RNA samples were extracted using a MagaBio Plus Virus DNA/RNA Purification Kit (BIOER Co., Hangzhou, China) and an NPA-32P automatic nucleic acid extractor (BIOER, Hangzhou, China). PRRSV RNA was detected using a commercial Quadruple Real-time Fluorescence RT-qPCR Kit (GuanMu Bio GM11076, Changsha, China) for PRRSV-2, including the North American, Highly Pathogenic strain, NADC34 like strain, and NADC30 like strain by differential diagnosis via the M and NSP2 genes with the ABI 7500 instrument (Thermo Fisher Scientific, Boston, MA, USA). The cut-off value was set at a cycle threshold (CT) of 40.

### 2.4. Exponentially Weighted Moving Average Method

Daily production data, including the number of abortion, off feed, low appetite, and dead sows was analyzed by using EWMA control method to recognize the deviation corresponding to alarm points. The EWMA smoothing parameter lambda (λ) and the sigma were set as 0.4 and 3.0, respectively which were referred to previous study [19]. The production parameters for 106 consecutive days of data in the PRRSV II-vx status were used to set up the baseline. The upper control limit (UCL) was set at three standard deviations above the process mean, and the lower control limit (LCL) was set at three standard deviations below the process mean. The EWMA detects an abnormal sign outside the UCL at the time of RT-qPCR testing and presents a positive result; this is classified as a true positive (TP). Conversely, when the EWMA detects a UCL abnormality, the RT-qPCR test of the aborted sows is negative and designated as a false positive (FP) [11]. The EWMA model was implemented using Minitab 19 software (Minitab LLC, State College, PA, USA).

### 2.5. Statistical Analysis

To analyze the deviations in various daily production indications at II-vx, I-A, and I-B statuses to assess which specific indication was more suitable for PRRSV monitoring, each daily data type of EWMA at the three statuses was evaluated using Kruskal-Wallis tests, followed by Dunn’s multiple comparison tests. The asterisk (*) indicates a significant difference between the two statuses for each data type (* *p* < 0.05, ** *p* < 0.01, *** *p* < 0.001, and **** *p* < 0.0001). “ns” means no significant difference. The RT-qPCR detection rates of the three different types of abortion samples were compared using the Cochran Q test, and then each two-sample combination sample detection rate was compared using McNemar’s test.

Data were analyzed using SPSS Statistics software (version 26.0; IBM Corp., Armonk, NY, USA) and visualized using GraphPad Prism 10.1 (GraphPad, San Diego, CA, USA).

## 3. Results

### 3.1. Utilize EWMA to Early Detect PRRSV Outbreak by Monitoring Four Production Parameters

**Figure 1:**
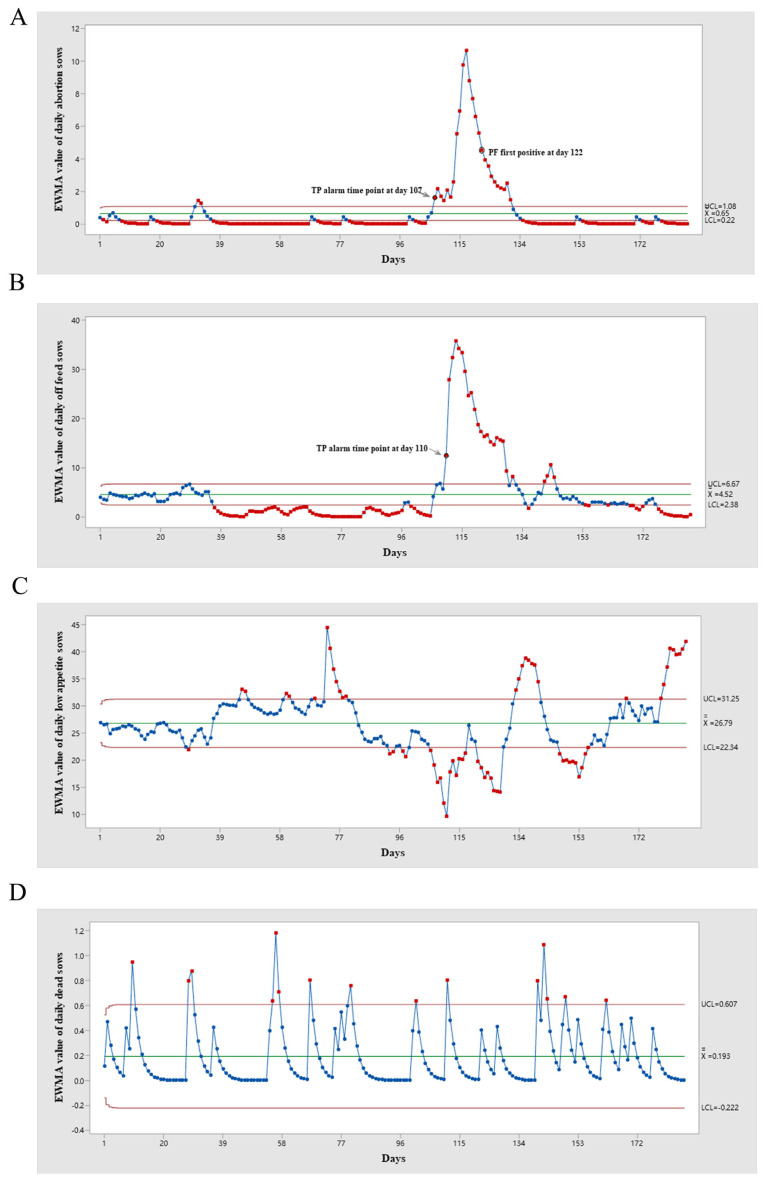
Exponentially weighted moving average (EWMA) charts of different daily production parameters. EWMA charts of daily abortion (**A**), off feed (**B**), low appetite (**C**) and dead (**D**) sow were monitored respectively. The true-positive (TP) alarm time point of efficient daily data was a point out of the upper control limit (UCL), which was indicated with an arrow in the figure. All the points out of the UCL without arrow indicated were false positive (FP) alarm time points.

**Figure 2:**
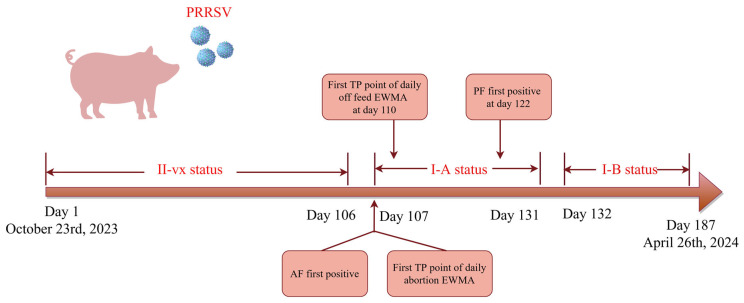
Roadmap and timeline for Porcine reproductive and respiratory syndrome virus (PRRSV) monitoring in the sow farm.

Different alarm time points were determined using the EWMA method, including abortion, off feeding, low appetite, and dead sows. There were two FP alarm points on days 32 and 33, and also twenty-five true-positive (TP) points from daily abortion sow monitoring (Figure 1A). There were twenty-five TP points and no FP points in the EWMA chart of the daily off-feed sows (Figure 1B). The first TP alarm points for daily abortions and off-feed sows appeared on days 107 and 110, respectively. Before and during the PRRSV outbreak, the daily low-appetite and dead sows did not exhibit any TP alarm points; however, some FP points emerged. There were 30 and 15 FP points for the daily low appetite and dead sows, respectively (Figure 1C,D). According to routine farm PRRSV monitoring, the first RT-qPCR-positive PF sample was detected on Day 122 (Figure 1). The timelines of the different PRRSV monitoring methods are shown in Figure 2.

### 3.2. Comparison of Each Daily Production Parameter by EWMA in Different PRRSV Statuses

**Figure 3:**
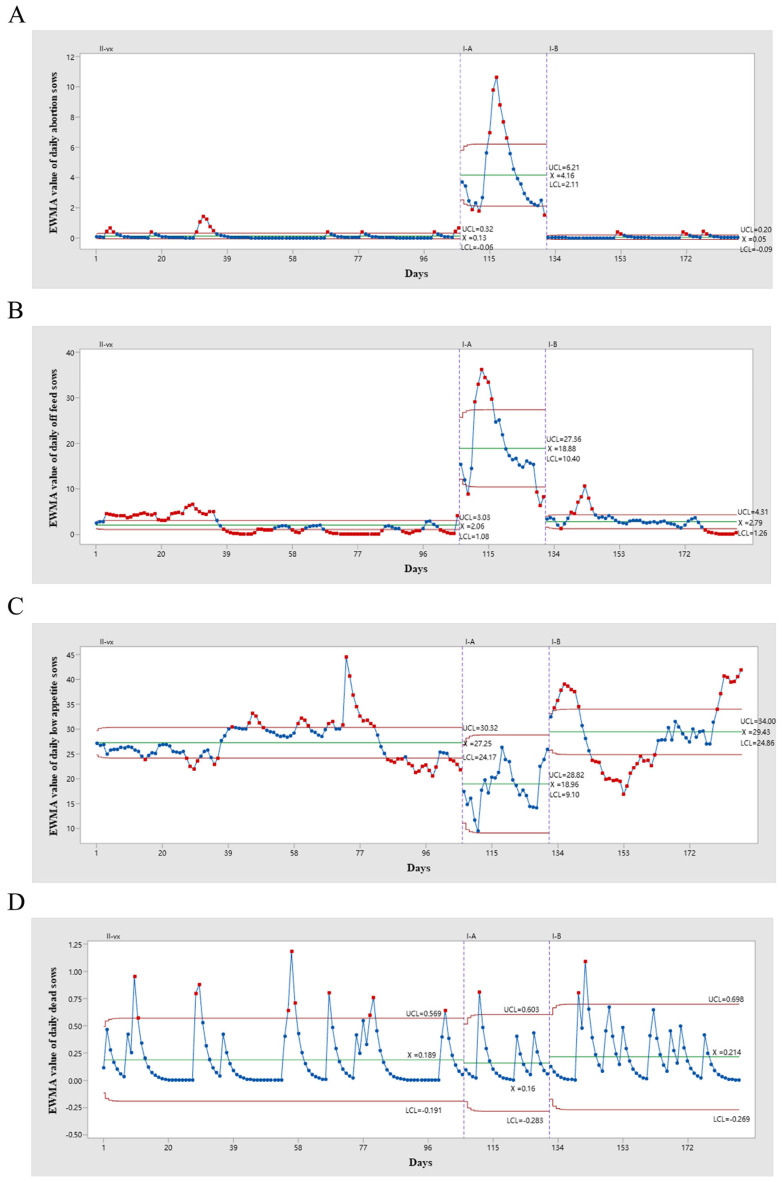
The comparison of each daily production parameter under different PRRSV statuses were conducted using EWMA method. The EWMA comparisons of daily abortion (**A**), off feed (**B**), low appetite (**C**) and dead (**D**) sows in the II-vx, I-A and I-B status were presented respectively.

**Figure 4:**
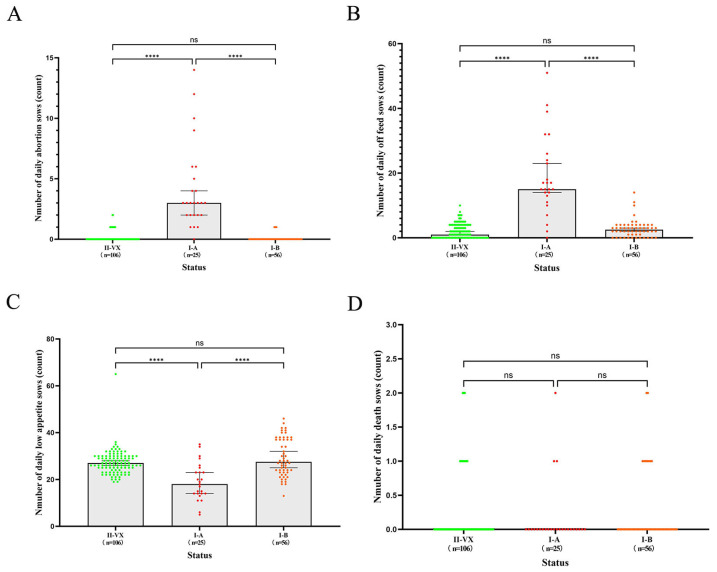
The comparison of each daily production parameter of EWMA under different PRRSV statuses were conducted by Kruskal-Wallis tests, followed by Dunn’s multiple comparison tests. The comparisons of the EWMA of daily abortion (**A**), off feed (**B**), low appetite (**C**) and dead (**D**) sows in the II-vx, I-A and I-B status were presented, respectively. The asterisk (*) indicates significant difference between each two status of every data type (**** *p* < 0.0001). “ns” means no significant difference.

The results indicated that the average number of daily abortions calculated by the EWMA under different statuses was 0.13, 4.16, and 0.05, respectively (Figure 3A). There was a significant difference in daily abortion sows from status II-vx to status I-A (*p* < 0.01), and I-A to I-B (*p* < 0.01), whereas no significant difference from II-vx to I-B (*p* = 0.99) (Figure 4A). In addition, the average values of daily off feed sows were 2.06 in status II-vx, 18.88 in status I-A and 2.79 in status I-B (Figure 3B). There was a significant difference from status II-vx to I-A (*p* < 0.01), and I-A to I-B (*p* < 0.01), but no significant difference from II-vx to I-B (*p* = 0.35) (Figure 4B). Average values of low-appetite sows were 27.25 in status II-vx, 18.96 in I-A and 29.43 in I-B (Figure 3C). There was a significant difference from status II-vx to status I-A (*p* < 0.01), I-A to I-B (*p* < 0.01), but no significant difference from II-vx to I-B (*p* = 0.83) (Figure 4C). The average values of daily dead sows were 0.189, 0.160, and 0.214 for statuses II-vx, I-A, and I-B, respectively (Figure 3D). Daily dead sows among the three groups showed no significant differences (Figure 4D).

### 3.3. PRRSV RNA Detection in Three Sample Types from Abortion Sows

Three different sample types were collected from 85 aborting sows during the PRRSV outbreak. Seventy-nine aborted sows were verified as PRRSV positive when at least one sample type was positive. Sample types had a significant difference in the detection rate (*p* < 0.01), that from 43.04% (95% CI 32.69–54.03%) in AF, 65.82% (95% CI 54.85–75.33%) in TBS to 74.68% (95% CI 64.11–82.97%) in the OS sample. The detection rate in AF samples was the lowest. There was no significant difference in the detection rate between OS and TBS; however, there was a significant difference between OS and AF (*p* < 0.01) and between TBS and AF (*p* < 0.01). The odds ratio of PRRSV detection in OS was 1.5 times (95% CI 0.8–3.1) and 3.9 times (95% CI 2.0–7.8) higher than that of TBS and AF (Table 1).

There were six negative reactions for both AF and OS. Seventeen negative reactions were observed in both the AF and TBS samples (Table 2). The detection rate of combination group of AF and OS samples was 92.41% (95% CI 84.41–96.47%), AF and TBS samples were 78.48% (95% CI 68.21–86.11%). There was a significant difference in the detection rate between the combination group of AF and OS with AF and TBS (*p* < 0.05). The odds ratio for PRRSV detection in the combination group AF and OS was 3.3 times (95% CI 1.2–8.8) higher than group AF and TBS (Table 3).

## 4. Discussion

Statistical process control (SPC) has been employed for years to detect process changes in swine production [19]. The EWMA method was selected because it offers greater sensitivity and specificity for detecting smaller deviations than other SPC methods [20]. This method can be easily implemented using the Minitab 22 software and does not require normally distributed data. Moreover, the EWMA method emphasizes short-term changes and identifies deviations in the production data, which is highly useful for minimizing the influence of previous data [21]. Once a new PRRSV is introduced into a herd, production indicators are affected. Therefore, different indicators should be utilized to fully and early detect PRRSV outbreaks. Weekly aborted sows and pre-weaning mortality have proven to be better indicators, with the highest detection rates compared to neonatal losses, dead sows, and off-feed sows [11]. Few studies have used daily production data for early detection of PRRSV outbreaks. Data on weekly abortion counts were collected and analyzed at the end of each week, whereas daily abortion counts were gathered and examined daily. Anomalies in weekly counts may emerge before weekends, making daily abortion data more sensitive. In this study, EWMA of daily production data was used for early detection and the results were compared with those of PF samples to detect PRRSV outbreaks.

Daily data on aborted and off-feed sows were both effective parameters for detecting PRRSV outbreaks using the EWMA. The results of this study are similar to those of previous studies that have used weekly abortion data [5]. The TP alarm time points for daily abortion and off-feed sows detected by EWMA were 15 and 12 days earlier, respectively, than when the RT-qPCR of PF turned positive. This may be associated with the first infection occurring in the third trimester of gestation in this study. Thus, the TP alarm point for daily abortion sows was approximately two weeks earlier than the PF-positive result. When the NADC30-like strain was first introduced into a breeding herd, pregnant sows initially exhibited clinical symptoms of off-feed and abortion, whereas lactating sows and piglets remained PRRSV-negative at that time. Many days later, positive PF results were observed when the infected gestating sows were farrowed. Another report indicated that the parameter with the highest detection rate in breeding herds was abortions, with the EWMA alarm point occurring, on average, 4 weeks earlier than the PF positive result [11]. The EWMA monitor for the number of sows with low appetite and dead sows did not detect PRRSV at an early stage. Before the outbreak, only AF samples from aborted sows and PF samples from piglets were collected for PRRSV monitoring, so low appetite and dead sows were not sampled to test RT-qPCR before day 107 to identify whether alarm points out of the UCL from daily low appetite or dead sow charts were TP or FP. However, PF samples from piglets born in batches with abnormal alarm points tested negative. Therefore, the alarm points outside the UCL from the daily low appetite or dead sow charts were FP before the outbreak. During the PRRSV I-A status, the relatively low number of sows with low appetite might have been due to a rapid increase in sows going off the feed; therefore, no alarm points appeared. Although some OS samples of low-appetite sows were positive by RT-qPCR, no TP alarm point appeared in the EWMA chart for daily low appetite. In I-B status, the RT-qPCR test of low-appetite sows was negative; therefore, some alarm points were FP. All RT-qPCR tests of dead sows after the outbreak were negative; therefore, the alarm points outside the UCL were FP points. The failure to detect a new introduction of the virus early based on the number of dead sows may be due to the low mortality rate caused by the NADC30-like wild strain in this study. In China, most NADC30-like strains exhibited moderate pathogenicity [22,23,24]. Additionally, most sow farms in China are routinely vaccinated with PRRS MLV, such as commercial PRRSV-modified live vaccines VR2332 [24], CH-1a [25], R98 [26], and TJM-F92 [27], which could partly provide protective efficacy against NADC30-like strain challenge. In this study, no sows that aborted following PRRSV infection died during the I-A and I-B stages, suggesting that PRRS MLV may provide partial cross-protection. Thus, the daily number of dead sows was not an effective predictor of the PRRSV outbreak in this case. Wild-type PRRSV strains with different virulence levels exhibit varying clinical symptoms. Although NADC30-like strain infections do not usually cause sow death under field conditions, some recombinations of NADC30-like and NADC34-like strains can lead to low mortality [28]. The EWMA method for monitoring the daily number of dead sows may be useful for early detection of highly virulent PRRSV strains. In another study, 12 simulated PRRSV outbreaks were used as a disease introduction scenario to attempt the early detection of unexpected trends in production data. The results showed that, compared to the weekly number of abortions (44%) and the weekly number of dead sows (4%), the weekly number of weaned piglets had the highest indication rate of 50% when using EWMA [29].

Abortion is a prevalent concern in swine production, and numerous factors can contribute to sow abortion [30]. Samples from aborted sows were collected by RT-qPCR to diagnose and determine whether reproductive failure was related to PRRSV wild-type strain infection. Fetal samples were used to test for vertical transmission, but necropsy on-site was forbidden on farms because of biosecurity issues. The AF is convenient and easy to collect. In field practice, AF samples showed the lowest detection rate. This may be related to the fact that not all fetuses were vertically infected with PRRSV, as well as an insufficient number of fetuses sampled by the staff. To improve the detection rate of PRRSV in aborted sows, the OS sample should be collected in combination with the AF sample. The combination of AF and OS samples showed a significantly higher detection rate than the combination of AF and TBS samples. PF is widely used to monitor the dynamics of PRRSV circulation in herds [11], and the limitation of PF is delayed detection if the introduction occurs in the gestation barns. Therefore, it will be helpful to complement the added detection from aborted sows with the swift detection of PRRSV. In future monitoring systems, a combination of AF and OS samples should be collected from abortion sows for early PRRSV detection.

The combination of EWMA of daily abortion data and molecular diagnostic methods is novel for the early detection of PRRSV outbreaks. This study was conducted on only one commercial sow farm, which limits the general applicability of early diagnosis of PRRSV outbreaks using EWMA combined with molecular diagnosis. Therefore, more farms should be involved in future production practices to verify the universal applicability of this method. Additionally, this study did not test for other potential co-infecting pathogens that could cause sow abortion. The use of daily abnormal production data EWMA as a proxy for PRRSV has not yet been fully established, and more research is needed to verify this. In different sow farms in China and other regions with different viral lineages, vaccine strains, and immunization programs, the application of this method for early diagnosis of PRRSV outbreaks has promising prospects.

Once a combination of methods can be verified and applied for the early detection of PRRSV outbreaks, under the current scale of tens of millions of sows in China, if it can provide a two-week warning before the outbreak of PRRSV, early intervention can be carried out through emergency immunization with PRRS MLV to reduce susceptible sows [31], using antibiotics such as tilmicosin to prevent secondary infections [32], or treatment with traditional Chinese medicine [33,34,35]. This will be of great significance in reducing the direct economic losses caused by PRRSV infection, such as abortions, stillbirths, and mummified fetuses, as well as in early immunization and culling to deliver PRRSV-negative weaned piglets. If this method can provide an early warning of PRRSV, it will serve as a reference for the early warning of other major viral diseases that cause abortion in sows.

## 5. Conclusions

Employing the EWMA to monitor daily production parameters proved to be a viable approach for the early detection of PRRSV outbreaks, especially when considering the number of aborting sows. In addition to the early detection of PRRSV outbreaks using the EWMA method, collecting AF and OS samples from aborting sows prior to conducting the RT-qPCR test is a complementary approach for the early detection of PRRSV. If a combination of methods is validated and implemented, it will significantly help reduce outbreak-associated losses.

## Figures and Tables

**Table 1:** Detection rates of Porcine reproductive and respiratory syndrome virus (PRRSV) in three sample types from abortion sows by RT-qPCR.

Sample Type	PRRSV Positive Number	PRRSV Negative Number	Total	Detection Rate (95% CI)	Odds Ratio (95% CI)
OS	59	20	79	74.68% (64.11–82.97%) ^a^	1 (reference group)
TBS	52	27	79	65.82% (54.85–75.33%) ^a^	0.65 (0.32–1.27)
AF	34	45	79	43.04% (32.69–54.03%) ^b^	0.26 (0.13–0.50)

^a,b^ Different letters indicate significant differences in PRRSV detection rates (McNemar’s test, *p* < 0.01).

**Table 2:** McNemar’s test between the matched abortion sow samples.

		OS Sample		Total	*p* Values			TBS Sample		Total	*p* Values
		Pos	Neg					Pos	Neg		
AF sample	Pos	20	14	34	0.001	AF sample	Pos	24	10	34	0.004
	Neg	39	6	45			Neg	28	17	45	
Subtotal		59	20	79		Subtotal		52	27	79	

**Table 3:** Detection rate of PRRSV in different combination group samples from abortion sows.

Sample Combination	PRRSV Positive Number	PRRSV Negative Number	Total	Detection Rate (95% CI)	Odds Ratio (95% CI)
AF + OS	73	6	79	92.41% (84.41–96.47%) ^a^	1 (reference group)
AF + TBS	62	17	79	78.48% (68.21–86.11%) ^b^	0.30 (0.11 -0.81)

^a,b^ Different letters indicate significant differences in the PRRSV detection rates among different sample types (McNemar’s test, *p* < 0.05). A positive sow was defined as one with at least one positive sample.

## Data Availability

No new data were created or analyzed in this study. Data sharing is not applicable to this article.
